# The Future Citizen

**Published:** 1917-03-31

**Authors:** 


### SOME PROBLEMS OF TO-DAY.

### I. The Future Citizen.

It is time that all women, and not least all nurses, learnt to "think Imperially." While certain questions must be fought out by our men on the battlefield, others whose menace we are only beginning to perceive must be faced at home in a warfare not less stern. Seen in the reflected light of war-fires, every hospital, every individual patient forms a fraction of that handicap of ill-health—that is, of inefficiency—which a nation needing all its strength has to carry. Rather let us say an *Empire*, since the debt due to our Colonies and Dominions must be repaid hereafter in terms of new life to fill countries that were only too empty before their depletion of men for the war, Canada with her two whites to a square mile, Australia with her one white to two square miles. And this new life needs to be given not only in quantity but in quality, strong in body where it leans to the animal, and in spirit where it reaches towards the Divine, as well as in mind, where skill and purpose develop. How to secure the needed measure of health and education for each child of the nation is our vital question, and when we come to face it squarely we find that while in 1870 we admitted—tardily enough—that every child had a right to be educated to take his place in a civilised community, we have scarcely recognised his primary right to life itself.

"It has taken a European war to convince us of the value of our children," wrote Mrs. Pember Reeves lately. It is only when the man—and the woman—in the street, Monsieur and Madame de la Rue, as someone has neatly phrased it, study the figures which have been year by year collected by our 1,800 sanitary authorities, and then pigeon-holed, that we learn how this precious asset has been wasted. By whom? The poor, overworked mother who has been actually ready to welcome "churchyard luck" needs to be awakened from the indifference of ignorance, but not more than the landlord who takes rent for her slum home, or the local Council responsible for its health conditions. Even if we could admit the desirability of wasting human babies like herring-spawn, we have now to learn that death is not the worst thing. Sir George Newman states that of 6,000,000 children on our elementary-school benches 1,000,000 are "so physically and mentally defective or diseased as to be unable to derive reasonable benefit" from their costly education. But "it is the child, and not his acquired accomplishments, which is of primary value to the nation—the living, unmaimed, normal child. . . ."

Now, what are the facts? In 1880 the average death-rate for England and Wales of infants under one year was 153 out of 1,000; in 1909 it was 100; in 1916 it had fallen to ninety-one; showing the immense wastage to be preventible. Further, the results of careful study show that the fall is great and immediate where organised effort is made for the instruction of mothers, especially by the establishment of infant-welfare centres and mother-schools. Thus, while Tunbridge Wells, most favourably situated, shows a reduction to 72.7, Hornsey, a purely urban district, has brought the figure down to 56.6. It is, indeed, a curious detail that a better average of health may often be found among children of slum districts, who are allowed to play in the street, than among those of a rather superior neighbourhood, who are kept in stuffy rooms until formally taken out walking.

When we realise that the reduction from 153 deaths in 1,000 births to 100 means a saving for England and Wales of 34,000 lives, we want to ask two questions: Are means being taken to effect the full possible saving? and are these children effectively saved, not merely dragged past the jaws of death to pine slowly, or to fill feeble-minded and cripple Homes, a constant burden to the family or the State? The answer to both depends on the effort made at once. The training of schoolgirls, even if their stay at school is prolonged by coming legislation, is hope for the future. For the immediate need we have 800 schools for mothers, 700 being affiliated to the new Central Institute of Mothercraft, while even in London, out of twenty-nine boroughs, only nine have infant-welfare centres. Beginning with Dr. Miele's first foundation at Ghent in 1901, and spreading quickly to seventy-seven in Belgium and over 1,000 in France, the School for Mothers has now obtained official recognition here by the Government undertaking to pay up to 50 per cent. of its expenses. Obviously, legislation for numbers is not the main thing; it is the work of individuals for individuals alone that can save the nation's children. When, for instance, Dr. Broadbent, during his mayoralty at Huddersfield, offered £1 to every child born during that time which lived through its first year, and later summoned a gathering of prize-winners, both quantity and quality were remarkably high. There is no sadder sight for a nurse working among the poor than to see the baby that weighed 8 lb. at birth shrivel at a year to a peevish thing of less than double the weight, afflicted by chronic cold or diarrhœa, or at three years old to find it cannot turn the scale at 25 lb. The Local Government Board circular sent in spite of war to municipalities, inviting them to increase their work for infants and mothers, is a sign of the times, and it is certain that many important and well-paid posts will open for those nurses who will turn their attention during or after training to this most important study, the preservation and healthy development of the future citizen.

